# Analysis of Social Science Research Into Cancer Care in Low- and Middle-Income Countries: Improving Global Cancer Control Through Greater Interdisciplinary Research

**DOI:** 10.1200/JGO.18.00045

**Published:** 2018-06-15

**Authors:** Carlo Caduff, Mac Skelton, Dwaipayan Banerjee, Darja Djordjevic, Marissa Mika, Lucas Mueller, Kavita Sivaramakrishnan, Cecilia Van Hollen

**Affiliations:** **Carlo Caduff**, King’s College London; **Marissa Mika**, University College London, London, United Kingdom; **Mac Skelton**, The Johns Hopkins University, Baltimore, MD; **Dwaipayan Banerjee** and **Lucas Mueller**, Massachusetts Institute of Technology; **Darja Djordjevic**, Weatherhead Center for International Affairs, Harvard University and Wits Institute for Social & Economic Research, University of the Witwatersrand; **Kavita Sivaramakrishnan**, Columbia University, New York, NY; and **Cecilia Van Hollen**, Woodrow Wilson International Center for Scholars, Washington, DC.

## Abstract

This analysis lays a framework for greater collaboration between the cancer community and social scientists in both research and policy. We argue that the growing cancer burden that low- and middle-income countries face is raising social, political, and economic challenges of global cancer that require interdisciplinary research beyond the traditional biomedical-clinical nexus. First, we briefly review some of the most important existing social science studies that have addressed cancer in low- and middle-income countries, including the main methods, approaches, and findings of this research. Second, we give an overview of recent interdisciplinary collaborations between social scientists and oncologists and demonstrate how qualitative research can help us to understand the distinct challenges of cancer care in low- and middle-income settings. Finally, we identify key areas for future collaboration and suggest possible paths forward for cancer research and policy that involve social science.

## INTRODUCTION

The scale of the global cancer burden and the unique social, political, and economic challenges that this poses have given rise to the demand for a new approach to policy that embraces social science. By drawing on research in India, Iraq, Kenya, Lebanon, Rwanda, and Uganda, we analyze how social science has contributed to the practical understanding of trajectories and solutions to global cancer control (Table 1). Only recently have social scientists begun to turn their attention to the cancer burden in low- and middle-income countries. In this analysis, we focus specifically on the contributions of anthropologists, sociologists, and historians to the social science literature while recognizing that other social science disciplines, such as economics, psychology, geography, and political science, have also made important contributions to the study of cancer. This brief review is based on our knowledge of the social science literature on cancer around the world. We do not consider ours to be a comprehensive literature review, although such an endeavor would be worthwhile. What follows is a critical review that highlights some of the most significant publications.

**Table 1:**
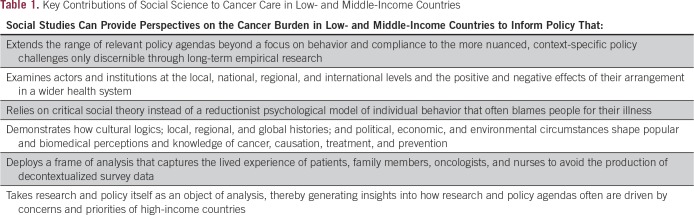
Key Contributions of Social Science to Cancer Care in Low- and Middle-Income Countries

Anthropologists, sociologists, and historians who study cancer in low- and middle-income countries draw from research with patients, family members, physicians, and nurses and observation of hospital settings. They examine the history and politics of policy making and changes in social attitudes over time and provide comparative accounts across societies that reveal common experiences. We have broken these studies into two categories: care-seeking strategies and interpretations of illness and practices among oncologists and nurses.

## CARE-SEEKING STRATEGIES AND INTERPRETATIONS OF ILLNESS

These social science studies attempt to draw connections between patient understanding of cancer and care-seeking strategies. Anthropologists, sociologists, and historians ask: How do patients interpret the causes of cancer and make sense of their illness, and what leads to changes in attitudes? How do interpretations of illness affect care-seeking strategies? How do care-seeking strategies interact with conditions of social, emotional, and financial duress? The many nuanced interconnections among these questions require a methodology that gives patients and family members a forum to articulate their experiences in their own terms. Consequently, most social scientists build in-depth case histories through long-term research in local settings. Many conduct multiple interviews over an extended period to track shifts in how patients and family members interpret and strategize around the disease. Often, these studies reveal disjunctures and disconnections between patient understanding of disease and those of medical specialists. Invariably, these enrich the epidemiologic picture or challenge it.

For example, in 1994, Hunt^1,2^ conducted interviews with patients and family members in southern Mexico and asked, “Why do you think this person got sick?” In the answers she received from patients and caregivers, she noted a strong moral tone, including all manner of flawed behavior—adultery, hedonism, domestic violence, promiscuity—that invokes images of purity and danger, propriety and misconduct, and goodness and evil. Consequently, patients and family members may understand the healing process as dependent on identifying and eliminating moral ills in addition to chemotherapy, radiotherapy, and other medical interventions. The physicians in Hunt’s sample, on the other hand, approached cancer as solely a medical and technical problem. Hunt suggested a bridging of these perspectives to enhance therapeutic effectiveness. Such insights are critical for policies around access, with genuine uptake by local communities.

A number of other studies have explored similar questions. In an ethnographic study of a Chinese village, Lora-Wainwright^3^ examined cancer etiologies and their implications for therapeutic inaction. Mulemi^4-8^ conducted research in a cancer ward in Kenya to study how patients experience and make sense of biomedical treatments. Through her work in a hospital in Mumbai, India, Macdonald^9,10^ focused on visions of hope among breast cancer volunteers. Broom et al^11-14^ examined medical pluralism, experiences of stigma, and shared decision making among patients with cancer in Hyderabad, India. These studies go beyond a reductionist psychological model of individual behavior by providing us with a more realistic account of the complex relations between interpretations of illness and care-seeking strategies.

## PRACTICES AMONG ONCOLOGISTS AND NURSES

Although the understanding of patients’ socially and culturally inflected interpretations of illness falls within the expertise of anthropologists, sociologists, and historians, one might not expect social scientists to comment extensively on the practices of oncologists and nurses. What could a social scientist possibly know about medicine that medical practitioners do not themselves already know? Although oncologists and nurses frequently are aware of the distinct social, political, and economic forces that shape medical institutions and medical interventions, they do not always have the time to study these forces systematically amid the daily pressure to deliver care to a large number of patients.

Recent work has explored regimes of oncologic practice in low- and middle-income countries. Among the most prominent publications is Livingston’s book *Improvising Medicine*,^15^ which looks at the ways in which oncology practice in Botswana specifically and Africa generally differs from the practice of oncology in the United States and Europe. In the absence of state-of-the-art therapeutic and diagnostic equipment, Livingston shows how the oncologist in a Botswana oncology ward resorts to alternative modes of establishing a diagnosis and administering treatment. Oncologists in these settings must review both new and old medical journals to piece together treatment protocols with outdated technologies. An ever-growing number of patients puts an already overloaded infrastructure under pressure, which makes it difficult for physicians to provide patients with adequate care. Livingston asked, “What is the best way for an oncologist to proceed in such a setting?" She found that improvisation is not an exception, but a norm.^16^ Along with Livingston, several social scientists have shown that cancer is shaped as much by social, political, and economic conditions and shifting health care practices as it is by aberrant cellular mechanisms.^17^ Without an understanding of the experiences of oncologists and nurses in resource-poor settings, education and training initiatives can easily fall wide off the mark.

## INTERDISCIPLINARY COLLABORATIONS

Interdisciplinary collaborations between social scientists and oncologists have delivered important insights for global cancer control and provide a template for future work. We dissect out these types of studies along thematic and country lines.

### Treatment Access and Administration

Cancer has long been considered a disease of mainly high-income countries.^18^ However, historical research shows that cancer always has been a health issue in low- and middle-income countries. The current concern with cancer as a major cause of death and disability in low- and middle-income countries needs to be situated within longer histories of cancer and cancer care. Often forgotten is that hospitals, such as Tata Memorial in Mumbai, and other medical institutions in low- and middle-income countries have been providing specialized treatments for decades to patients with cancer, not only in their own populations but globally, as well, through international networks. Yet even in well-developed oncology centers, major challenges to accessing and administering treatment remain. We focus on treatment challenges in Uganda, Rwanda, Iraq, and India.

#### Uganda.

Since 2010, Mika^19^ has conducted research at one of the oldest sites that provides medical oncology services in sub-Saharan Africa, the Uganda Cancer Institute (UCI), which opened its doors in the late 1960s to treat Burkitt’s lymphoma and a range of solid tumors. Mika’s findings can be summed up in one sentence: Ugandan physicians, nurses, laboratory technicians, and palliative care specialists all exhibit tremendous creativity in times of a growing cancer crisis.

Mika began her work at UCI at a moment when senior leadership was particularly interested in excavating the rich history of the site. The collaborations with UCI have focused on archival preservation and have drawn on the history of cancer research and care to shape policy in the present. The collaboration also has created a space to reflect on the ethics of long-term ethnographic research in a hospital setting and how history can inform policy. UCI houses records on patients that date back to the mid-1960s. If preserved and made accessible, these would be an invaluable collection on the epidemiology and treatment of cancer on the African continent. By working with senior staff at UCI, Mika hopes to restore, digitize, and create an accessible database of these records in the next 2 to 3 years.

#### Rwanda.

Djordjevic’s^20^ research looks at national oncology in Rwanda, where she has been conducting research since 2010. During 2010, she witnessed the gradual beginnings of oncology in the form of a breast and cervical cancer prevention campaign launched by the Ministry of Health in collaboration with Partners In Health, a Boston-based nongovernmental organization (NGO) already substantially engaged with the public health sector infrastructure and care delivery. In 2012, these partners and others inaugurated the Butaro Cancer Center of Excellence, Rwanda’s first public cancer center, nestled in the hills of northern Rwanda near the border with Uganda. From the beginning, the center has been the product of significant transnational private-public partnerships. Treatment is delivered by young Rwandan physicians and American clinicians (most fresh out of residency) affiliated with Harvard University hospitals. Chemotherapy is paid for by Partners in Health, and on a monthly basis, the organization selects 7 to 12 patients who they send to the Nairobi Hospital in Kenya for radiation therapy.

What are the major insights to be gleaned from Rwanda’s public oncology program? One is that we need to pay close attention to how private and public are defined on the ground given the widespread prevalence of private-public partnerships for oncology in low- and middle-income countries. What remains compelling and unique is that despite major private contributions from abroad and heavy financial dependence on such contributions, Rwanda produces and exercises national ownership over the oncology program in a variety of ways. In certain respects, the program is a model for oncology in low- and middle-income settings, but a critical eye helps us to understand the limitations and strengths of various aspects of the model and how to apply these in Rwanda and beyond. Another is that oncology holds particular power for the Rwandan national imaginary given the technologically demanding treatments and modalities that cancer requires. Such understanding is important for the political dimension of cancer control and informs how externally supported developments of cancer care should be nuanced to suit local realpolitik.^21^

#### Iraq.

Skelton et al’^22,23^ research looks at cancer care in the context of war and mobility in Iraq, particularly in the northern provinces. They track the mobility and strategies of Iraqis with late-stage cancer as they move across provincial and international borders in pursuit of oncology care. This research explores how cancer is increasingly a disease that, particularly in the conflict settings of the Middle East, is managed across both domestic and international borders. Iraq’s once robust oncology infrastructure has faced 25 years of de-development as a result of United Nations sanctions (1991 to 2003) and insecurity (2003 to present). This process has generated new strategies of care seeking and an emerging regional geography of care. Patients manage war-induced deficiencies in care by piecing together treatments across multiple domestic provinces and international sites. This interprovincial sojourning is largely a function of the fact that Iraqi public hospitals negotiate the problem of pharmaceutical shortages and the mandate to provide universal care by rationing limited doses per patient, which means that one is forced to complement these provisions either through movement between provinces or internationally. And yet, this movement itself is complicated or inhibited by security/checkpoint policies and threats of violence.

In addition to conducting his own research, Skelton has co-led two collaborative projects with oncologists, one based in Lebanon and the other based in Iraq. In Lebanon, he has collaborated with a team of oncologists based at the American University of Beirut Medical Center in a mixed-methods qualitative/quantitative study on the financial hardships of Iraqi patients with cancer who undergo treatment in Lebanon. In Iraq, Skelton is collaborating with oncologists based at three public cancer centers to investigate the pathways of cancer care for displaced patients.^24^ This research looks at the networks and pragmatics that enable families and patients to access the out-of-pocket resources needed to manage the enormous financial burdens of cancer care amid conflict conditions that already deplete incomes and destroy livelihoods (Skelton et al, manuscript submitted for publication).

#### India.

Sivaramakrishnan’s work, which is based on research in the northern state of Punjab, traces the mobility and migration that patients and health practitioners have struggled with both past and present because care is clustered in urban metropolises that require travel and resources. Sivaramakrishnan also explores the fractured networks of mobility involved in consulting for care and reconciles diverse therapeutic strategies among plural systems of medicine from homeopathic pharmacies to Ayurvedic clinics to rural medical practitioners. Currently in India, cancer campaigns have been revised and recast since the formulation of state and national policies, but still, class and epidemiologic hierarchies are frequently conflated, and specific cancers are still associated with certain populations (eg, cervical, oral). Differentiation of these cancers from the more upper-class cancers of the breast persist. Sivaramakrishnan stresses that some of the biggest challenges to deepening cancer-related research and affordable care in low- and middle-income countries remains a reluctance among politicians, philanthropists, and the middle-class public to view cancer as an urgent and universal public health priority.

### Prevention and Screening

On the basis of fieldwork in Tamil Nadu, India, anthropologist Van Hollen^25^ (manuscripts submitted for publication) has explored perceptions of cancer causality, prevention, and treatment in response to government and NGO advocacy efforts to promote screening for reproductive cancers (particularly cervical and breast cancer) in low-income communities. Like many other countries, India faces a major problem in terms of early detection and treatment of cancer. Most people do not seek medical care for cancer until the disease has progressed to advanced stages. Until recently, screening interventions have been scarce and too costly (in terms of both time and money) for poorer populations. Fear and social stigma associated with cancer also have deterred early detection. In response to these societal conditions, global, national, state, and NGO institutions are actively engaged in developing programs to increase screening and early detection.

By contesting the notion that global public interventions disregard local sociocultural contexts,^26^ Van Hollen’s research has shown that public health planners take great pains to localize the educational messages to increase the use of screening and early detection. Too often, however, such culturally appropriate public health messages convey highly moralizing judgments. They imply behavior norms associated with sexual and reproductive practices, age of marriage, food, exercise habits, and tobacco use. By using social science methods that combine observation with qualitative, open-ended interviews with physicians, nurses, public health counselors, and people with low incomes who are the targets of these campaigns, Van Hollen has been exploring how these culturally appropriate messages may further stigmatize cancer and deter screening.

Social science research on cancer screening and prevention interventions around the world can help to avoid the pitfalls of moralizing discourses. Such studies are critical if global cancer prevention measures are to gain national political traction. These studies bring the voices of communities targeted by public health programs to the table. A shift away from individual causality can foster a more robust and politically challenging debate about the need for more regulation of the global production and consumption of potentially carcinogenic substances, which will add to the traditional econometric approaches.^27^

As Mueller’s historical research has shown, toxicologic studies often are based on factory and other workplace settings common in early 20th century Europe and North America. These studies often have been limited to animal experiments in highly controlled laboratory environments. Such studies may not capture the more complex relations of exposure in agricultural labor, including exposure pathways that are shaped by interactions of various chemicals, sunlight, rain, and other environmental conditions.^28^ Historical accounts have suggested that studies on specific exposures in low- and middle-income countries are urgently needed, especially because pesticides, naturally occurring carcinogens, and other toxic substances may behave differently in other environmental conditions and that such studies should take community concerns seriously because these might point toward previously unrecognized toxic exposures.

This approach would require better funding for occupational health programs and testing laboratories. Agricultural development programs, sponsored by international donors, will need to take toxic exposure and cancer prevention into consideration when designing interventions. Social science research can help with understanding assumptions in toxicologic knowledge and the limits of implementation of legal regulations. With that, the social sciences can help to broaden cancer prevention from being limited to immediate biomedical interventions to addressing the problem of toxic exposure to carcinogenic substances more generally.

### Palliative Care

Banerjee’s research on terminal cancer care in India highlights the need to strengthen palliative care infrastructures. Although cancer incidences are higher in high-income countries, the burden of mortality falls disproportionately on low- and middle-income countries. To understand the social lives of those who seek palliative care in Delhi, India, Banerjee conducted 1 year of ethnographic interviews and observations with cancer care NGOs and hospitals as well as in the homes of low-income patients with cancer and their families. His research has revealed and answered an important ethical conundrum that faces global cancer care: How might public health practitioners aid urban poor patients in their time of dying without losing sight of the need to expand access to treatment and pathways to survival?

The past two decades have witnessed the development of at least two contrasting models. In Delhi, the NGO CanSupport has pioneered the delivery of expert care by teams of professionals to the homes of urban poor patients. In Kerala, a state with a long history of communist mobilizations, groups such as Pallium India and the Neighborhood Network in Palliative Care have advocated a community-based approach by training large groups of volunteers to deliver care to their neighbors and kin. These palliative care efforts have revealed three things. First, although low- and middle-income countries lag in cancer care, they have been sites of innovation with lessons for high-income countries, especially resource-rich countries like the United States that have mobilized around tertiary care and expensive cancer technologies rather than around accessible end-of-life care. Second, the development of cancer care in low- and middle-income settings confronts difficult choices between access and expertise. A crucial task for palliative care work (and indeed for cancer care in general) is to understand existing networks of care and to adapt and articulate with them organically, which involves an understanding of social worlds of support as both sources of stigma, violence, abuse, and neglect and sources of potential help and support. To this end, Banerjee collaborated with palliative care physicians in northern India’s largest public health facility: the All India Institute for Medical Science. Together, they published a clinical audit of the palliative care unit’s pain assessment procedures. This collaborative work across the social and biomedical sciences emphasized the need to pay closer attention to psychosocial etiologies of distress in clinical practice.^29^ Third, palliative care organizations in low- and middle-income countries share a fundamental concern: the availability of effective analgesia. Oral morphine and other opiate-based painkillers are fundamental to the effective treatment of cancer pain, yet < 3% of patients with cancer in India have access to oral morphine. Of note, this problem is not one of scarcity. Although India produces > 90% of the world’s licit opium, opiates rarely have been available within the country because of strict drug legislation. The efforts of palliative care professionals has led to the relaxation of rules in some Indian states, but physicians from higher-class backgrounds continue to fear addiction among lower-class patients and rarely prescribe the drug. Here we see again that framing the problem of cancer in low- and middle-income countries in terms of scarcity elides the complex social, cultural, and political factors that promote and/or inhibit cancer care.

## DISCUSSION

Social scientists are committed to studies that foreground the narratives of a wide range of stakeholders (ie, patients, family members, medical practitioners at all levels and from many systems of medicine, researchers, policymakers) to give voice to the lived experiences, motivations, and constraints of all who are touched by cancer and involved in cancer care. This approach humanizes and adds richness to our understanding of cancer in heterogeneous and complex settings across low- and middle-income countries. Social science research favors studies that span extended periods for continuous observation and engagement. It enables interdisciplinary collaborations and generates unique analyses that other methods cannot offer and that clinicians often cannot conduct given the pressing demands of delivering medical care. A significant benefit is that social scientists usually return to the same research site. The personal connections and experiences that grow over time enable social scientists to have a thorough understanding of changing conditions that affect oncology.

Social science research not only reveals the miseries and stigma of patients, the lack of access to medical care, and the fraught social negotiations of the sick and their families but also interrogates the quality and efficacy of the interventions extended and delivered to low- and middle-income countries. Cancer treatment demonstration programs often increase inequities within poor countries by establishing state-of-the-art cancer clinics that serve only a fraction of the population. The goal of social science research is not to undermine the global oncology equity agenda but to retain a deep analysis of risk-benefit equations, resource-intensive technologies, patient experience, and sustainability, which are crucial for well-informed policy.

A key question for policymakers is how to respond to the uneven ways in which medical institutions are evolving in a world that is increasingly marked by the privatization of health care. In places with no or little health insurance coverage, the responsibility of shouldering health care costs often is left to patients and family members as out-of-pocket and catastrophic expenditures. The global cancer community is increasingly aware of the financial burden of treatments and the associated risk of exacerbating already-existing inequalities. To deliver accessible and affordable care for the benefit of patients, the global cancer community needs to move beyond the assumption that low- and middle-income countries must catch up with or mimic the oncology principles of high-income countries.

Social science research reveals assertions and assumptions about cancer and its causes and consequences through medical anthropology, medical sociology, history of medicine, and related fields in low- and middle-income countries. Interdisciplinary collaborations can then extend across the social and medical sciences and high-, low-, and middle-income settings to understand the limitations that these assertions and assumptions impose. Open discussion will help with developing more-effective and -comprehensive cancer policies that combine treatment, prevention, and palliative care for all. An urgent need exists for practice-informed social science research in countries where social, political, and economic inequalities are major determinants of poor cancer outcomes.^30,31^ National and international development priorities often do not fit well with the social, political, and economic conditions in low- and middle-income countries. National cancer control programs must be based on a robust understanding of local realities and lived experiences, which only social science can provide.

One of the reasons that social science approaches to the study of cancer have been historically under-represented in the cancer research literature is that the conclusions often emphasize local conditions; question basic assumptions; and call for social, cultural, and economic changes that are politically challenging. This reason should not deter social scientists from pursuing such studies. In fact, it should inspire us to redouble our efforts and find ways to convey findings clearly to a broader audience in the interests of improving cancer care around the globe.
